# Walking groups in socioeconomically deprived communities: A qualitative study using photo elicitation

**DOI:** 10.1016/j.healthplace.2016.02.007

**Published:** 2016-05

**Authors:** Sarah Hanson, Cornelia Guell, Andy Jones

**Affiliations:** a Norwich Medical School, University of East Anglia, Norwich, Norfolk NR4 7TJ, United Kingdom; b MRC Epidemiology Unit and UKCRC Centre for Diet and Activity Research (CEDAR), University of Cambridge School of Clinical Medicine, Cambridge Biomedical Campus, Box 285, Cambridge CB2 0QQ, United Kingdom

**Keywords:** Walking groups, Social practice, Health equity, Public health, Photo-elicitation

## Abstract

Walking groups can benefit health but uptake is more likely amongst those who are socially well-situated and need them least. This study worked with a new walking group in a community in England with poor health and socio-economic indicators to understand non-participation and barriers to involvement. It used a qualitative approach. Participant generated photographs captured the physical and social environments in which they walked and these were used with semi-structured interviews to inductively explore walking group participation and the wider social context of walking. We found that prior to joining there were low expectations of any health benefit and walking groups were not viewed as ‘proper’ activity. The group format and social expectations presented a barrier to joining. Having joined participants described a developing awareness of the health benefits of walking. The shared sense of achieving health goals with others sustained the group rather than socialising, per se. We suggest that walking group participation is a complex social practice. Promoting walking groups as a social activity for this group of people may well have been counter-productive.

## Highlights

• Photo-elicited interviews explored physical and social environments for walking.
• Group walking was not viewed as effective exercise with health benefits.
• Group format and social expectations represented a barrier to these participants.
• The walking group was sustained by achievement of goals rather than sociability.
• Promoting walking groups as a social activity can be counter-productive.

## 1 Introduction

Physical activity has wide-ranging long-term health benefits (Reiner et al., 2013). Recent research has shown that the greatest gains to population health could come from inactive individuals becoming moderately active by exercising equivalent to just 20 min of brisk walking each day, reducing the risk of premature death by between 16% and 30% (Ekelund et al., 2015). Walking is a natural and safe form of exercise (Hootman et al., 2001). For most people it expends enough energy to be considered ‘moderate intensity’ activity. Furthermore, for individuals who are particularly unfit, walking at a pace of 3 mph can achieve activity that is of vigorous intensity and confer associated health gains (Kelly et al., 2011). Walking is therefore a sensible starting point for people overcoming inactivity (Murtagh et al., 2002). While exercise-based physical activity interventions appear to have only modest or short-lived success, promoting walking might appeal to the wider population as it does not require particular skill, equipment or a competitive nature. Walking schemes are encouraged at community level and may be used in Exercise Referral Schemes for those who are inactive and with health conditions (National Institute for Health and Care Excellence, 2014, Public Health England, 2014). Group walking has the potential to engage those who are interested in the outdoors, whether for leisure or as a health intervention. As outdoor walking group participation can confer both physiological and psychological multiple health benefits, with good adherence and few side effects they are a promising intervention as an adjunct to other healthcare, or as a proactive health promoting activity (Hanson and Jones, 2015).

However, while research shows that walking appears to be a popular form of physical activity across all socioeconomic groups in England, those more highly situated are nearly twice as likely to partake in recreational walking compared to those who are less well situated (46% compared with 25%) (Fox and Rickards, 2004). In walking interventions, uptake seems to be mainly by white, well-educated, middle aged women (Foster et al., 2011). Additionally, successful walking group recruitment is often judged by the numbers who join rather than those who would stand to benefit most (Matthews et al., 2012). As easily recruited participants tend to be those who already walk, recruiters are challenged about how to approach and persuade those who do not walk to walk often, especially in ‘hard to reach’ groups, such as the most deprived (Foster et al., 2011). Whilst walking groups improve health (Hanson and Jones, 2015) they also have the potential to widen health inequality if not well targeted (Foster et al., 2011, Ogilvie et al., 2007). This presents a need to understand how the health benefits of group walks can be ‘democratised’ to widen participation (Green, 2009).

Walking groups can be conceptually placed within social theoretical debates of public health’s focus on lifestyle behaviours. Coined as ‘new public health’ in the 1990s, social scientists problematized its narrow focus on lifestyle related prevention, which places the onus and responsibility on the individual to exercise control, be healthy, and become productive citizens (Lupton, 2003). The population is handed “biological responsibilities […] embodied in contemporary norms of health and practices of health education” (Rose, 2007, p. 133).

Public health agendas have somewhat shifted in recent years towards wider social and structural determinants and a growing recognition that the context of people’s lives needs greater consideration to reduce ‘lifestyle’ disease (Cohn, 2014). The life-course is subject to a range of influences and people are not merely ‘blank sheets’ awaiting and receptive to health promotion messages (Baum and Fisher, 2014, p. 215). Rather than being unaware of the risk, it is more likely that constraints in people’s their lives makes behaviour change difficult (Baum and Fisher, 2014). It is possible that walking groups fit into this more holistic public health agenda by providing structure and opportunities for physical activity rather than narrowly focusing on sports and exercise. For example, within public health and epidemiology, there is an increasing research field on the health benefits of green space, either as opportunities to be physically active in, or more generally as health enhancing spaces. This includes Gesler’s seminal work (Gesler, 1992) and more recently, work such as Gatrell (2013), Roe et al. (2013), and Bowler et al. (2010).

Without understanding both individual needs and life situations, there are potentially barriers created for those who have the greatest health need (Matthews et al., 2012). For walking, it appears to be particularly important to understand both the physical and social environments in which the behaviour takes place. It may be that for those people from more deprived backgrounds, rather than being a pleasurable leisure activity, walking may be their only available and affordable mode of transport. Consequently, walking may be burdensome or stressful, for example when walking with small children or in unsafe neighbourhoods (Bostock, 2001, Green, 2009). This might partway explain why in the United Kingdom, despite its past history of socialist walking clubs, rambling (walking in the countryside for pleasure) has a particularly middle class identity (Green, 2009).

Using a qualitative approach, this study worked with a newly formed walking group as part of a referral scheme in a place of social and health deprivation with participants with multiple health problems. The aim was to add to our understanding of non-participation in walking groups for particular social groups and thus how they can be more effectively promoted to target people in those communities who could benefit most. To do so this study was framed within a social practice perspective which aimed to highlight the social context of walking and walking group participation, rather than understanding it as an individualised health behaviour (Blue et al., 2014). Social practice theories have recently been applied by sociologists to understand health behaviours as sets of activities that are shared across time and place (Blue et al., 2014). Understanding walking as a social practice, this relatively recent approach to conceptualise behaviour change suggests that we need to understand more broadly, how health practices emerge, persist or disappear as shared practices (Blue et al., 2014). Bourdieu (1977, p. 86) made sense of such socially shared practices as ‘habitus’, which they defined as the, ‘subjective but not individual system of internalised structures, schemes of perception, conception and action common to all member of the same group or class’. Habitus includes knowing the right cultural codes and what works in different contexts and settings. It also refers to the values and expectations of particular social groups and the reactions of individuals within these groups in terms of behaviour that they see as reasonable and of common sense. When joining a walking group, the individual enters a new space or field. Each field has its own rules, coined ‘doxa’ by Bourdieu (1977). The individual brings to the group their habitus, which others in the group will evaluate and adapt to, thus the group is socially situated and evolving.

## 2 Methods

Semi-structured interviews were elicited using participant-produced photographs, as this method has previously been found to yield different insights and produce rich observational data (Guell and Ogilvie, 2013). This research focused on the factors that had influenced participation in a walking group and their perspective of how participation has impacted their health and wellbeing.

This study was given a favourable ethical opinion by the NHS NRES Committee South West-Exeter in June 2014. Participants were offered optional consent to their photographs being used by the research team in publications and presentations.

### 2.1 Setting and participants

The walking group is operated by East Coast Community Healthcare, NHS (East Coast Community Healthcare, 2015) in Great Yarmouth in the east of England. Great Yarmouth is a seaside town with built tourist attractions. It has deprivation that is higher than average and a health profile that is generally worse than the English average. For example, 29.7% of adults in this local authority are classified as obese, against an England average of 23%. Similarly, the under 75 years cardiovascular mortality rate is 92.6 per 100,000 compared to an England average of 78.2 (Public Health England, 2015). The district of Great Yarmouth has a population of approximately 97,000 inhabitants (Office for National Statistics, 2013). Socio-economically, by occupation, 51% are classed as decile 5–8 (lower supervisory, semi-routine, routine occupations and never worked) and it ranks in the highest decile of English districts for employment of unskilled and semi-skilled work (Office for National Statistics, 2013).

All participants were part of the UK National Exercise Referral Scheme (National Institute for Health and Care Excellence, 2014), referred by their family doctor to the physical activity (PA) trainer with a health need that would benefit from increasing physical activity. The scheme uses both a community gym option and outdoor walks run by the PA trainer who monitors participant’s health with anthropometric measures and quality of life questionnaires recorded at baseline, 6 weeks and at the programme end. The walks were developed and risk assessed by the PA trainer as safe with variety (in surfaces e.g. beach and concrete and also gradient) and the ability to do switchbacks and wider loops for those who were more physically able. At the initial consultation with a PA trainer, both gym and walking are explained and participants make an informed choice based on their physical (dis)ability and what they consider they are most likely to adhere to. The participants in the study chose to join the walking option, walking with the group for 12 weeks, once or twice per week for 50 min on each occasion with 5 min cooldown exercises at the end.

Participants had a range of both physiological and psychological health needs. One was of normal weight (BMI 24.4) and nine were overweight or obese (BMI 29.1–48.5). There was also diagnosed chronic obstructive pulmonary disease (COPD), type II diabetes, enduring mental health problems, depression and low mood. Most had multiple health problems that were affecting their quality of life. The participants were six female and four male. Three were aged 40–50, six were aged 50–70 and one was over 70 years of age. The participants were not of professional/managerial backgrounds.

This is a small scheme and therefore all those who started health walks between July 2014 and February 2015 were approached with a letter inviting them to participate. All 10 participants consented.

### 2.2 Photo-elicitation process and interview framework

Participants were given a disposable camera and simple instructions to capture images to represent what is helpful and unhelpful to walking in everyday life and positive and negative experiences of belonging to a walking group. Sensible care guidance in taking photographs in public places was explained. Disposable cameras were used as they require only straightforward training without expectation to produce high quality photographs or for participants to be embarrassed by what they have produced (Guillemin and Drew, 2010). Participants were encouraged to photograph a range of images and to avoid the social conventions of taking positive images (Guillemin and Drew, 2010). Participants returned the camera to the researcher (SH) approximately two weeks later for the photographs to be developed. Interviews were held a further two weeks later within National Health Service (NHS) premises familiar to the participants. Individual interviews were deemed most appropriate in anticipation of the participant making reference to their personal health. The semi-structured interviews used the photographs both for open-ended, participant driven elicitation and also as a basic interview guide. This ensured that relevant issues were covered but also enabled probing and development of issues pertinent to each individual and points the participant raised from their photographs.

At the beginning of the interview, participants spent a short time on their own familiarising themselves with their photographs, sorting them in a way that was meaningful to them. This enabled a more organised dialogue, with participants generally choosing to categorise photos as positive and negative and taking greater charge of the opening of the interview as they explained this. They then explained why they had captured their photographs and what the images represented. An additional interview guide was used to probe for further information or to elucidate areas that had not being discussed with the photographs. Two of the ten interviews were conducted as a pilot with discussion between SH and CG.

All interviews were conducted by a female doctoral student (SH). Participants were aware that this study formed part of a doctoral thesis. The researcher was trained in qualitative research techniques and interview skills with CG as the qualitative supervisor. Typically interviews took 45 min.

### 2.3 Data management and analysis

Interviews were digitally recorded and transcribed verbatim (by SH). Data was transcribed and analysed as it was collected and initial codes attached. Despite the small sample, during the latter interviews there was repetition of similar answers covered by the team-agreed coding scheme and saturation of emerging themes was felt to have been achieved to a satisfactory level (Ezzy, 2002). A process of funnelling transformed initial coding into categories. From this major themes were developed (Ezzy, 2002). Analysis was led by SH as the main researcher and monitored by regular meetings with both CG and AJ throughout the process. This enabled cross-checking of both emerging ideas and interpretation of the data. Management of the data was aided using NVivo 10. The study followed the consolidated criteria for reporting qualitative research (Tong et al., 2007).

## 3 Findings

Two participants were not confident in taking photographs and chose only be interviewed. All other eight participants used the cameras, collecting a total of 210 photographs. A broad breakdown of images represented is presented in Table 1.

The following represent major themes which emerged from the interviews and the images that participants generated. Themes are supported with illustrative quotes, with names as pseudonyms.

### 3.1 Places of everyday walking

Participants were specifically asked about their walking habits in everyday life and to capture images that represented this. This is a deprived neighbourhood with industrial features and urban spaces in need of general maintenance predominating. The majority of the photographs captured this, which was explained both positively and negatively. Roads, carparks and car parking in general were seen as negative and barriers to walking. These were viewed as being unpleasant to look at, difficult to navigate with pervasive traffic noise spoiling walking. However, these neighbourhoods also featured parks and cemeteries that participants highlighted for their positive aesthetics due to quieter spaces and attractive fauna and flora. Adding walking journey time to utilise positive spaces and avoid pavements and traffic noise was seen as desirable. Although traffic was viewed negatively, all participants made use of a car, bus or electric bike for household shopping.

Notably, the participants’ physical environment also included a beach, dunes, park and promenade, which presented a local leisure opportunity for all the participants and was utilised as the location for the walking group route. It sits as an 'edgeland’ to an urban area with small seafront hotels, fairground, an industrial harbour and off-shore windfarm. Although photographs of the seafront (beach and sand dunes) also captured urban features, these were not necessarily expressed as exclusively negative. For example, when presenting a picture of the sea and beach which included an off-shore wind-farm in the distance, one participant expressed that, 'I suppose it spoils the view’ but also that it did not particularly bother her (Tracy, aged 40–45 years). Promenades with benches were viewed as helpful resting places with concrete surfacing facilitating easier walking.

Many captured images and talked about the pleasure of previous walking for leisure throughout the life-course. This included nostalgic re-visiting and photographing places that had represented enjoyable walks. This included open fields and a riverbank, which had been enjoyable prior to ill health and ‘old age’ or used for walking a child in a stroller. Others valued man-created spaces, such an allotment and a fishing lake. These were appreciated for the pleasure of the outdoors as a hobby rather than as an opportunity for physical activity.

Others viewed walking quite differently and did little walking in everyday life. They captured images that did not represent places for walking, instead illustrating experiences of ‘non-walking’ habits.

*I never think oh I’ll get up and go out for a walk. I’ll sit in unless I’ve got a reason to go out. I haven’t been to the seafront. Before that [walking group] I hadn’t been down to the seafront for years. (Robert, aged 50–55 years)*

**Image 1:**
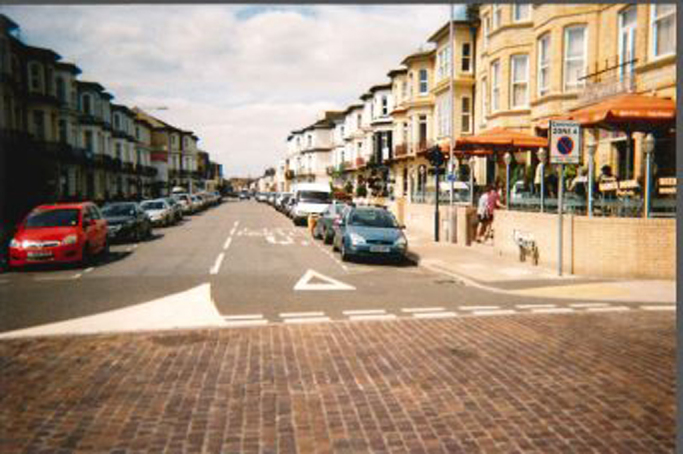
Photograph of their home street taken by participant from edge of beach.

To demonstrate this, Robert captured an image of his home location, taken from the edge of the beach to show its very close proximity (Image 1).

**Image 2:**
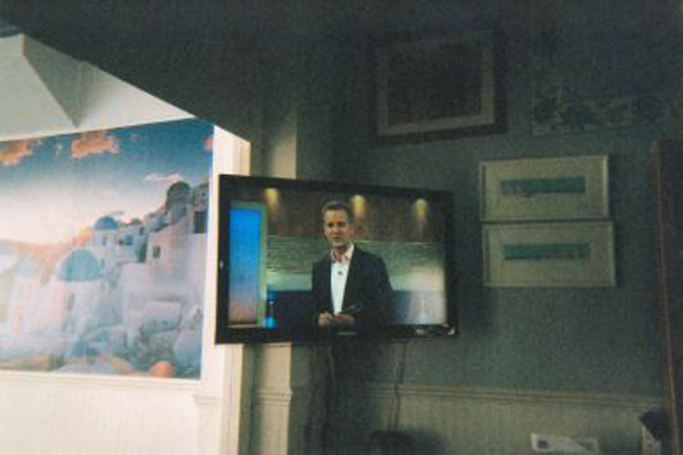
The world of this participant revolved around their living room.

Another participant photographed her car, sofa and television (Image 2) to explain her lack of everyday walking.

*I’ve got the car. What do I need to walk for? My sofa, it is very comfortable. I put my feet up, I’ve got my iPad, my TV, I mean what would I need to go out for? My best friend my iPad. I sat there this morning Christmas shopping on it so I haven’t got to go out for a walk up the shops, I’ll buy that. Yes, that’s my little corner, TV, iPad, sofa. (Brenda, aged 55–60 years)*

### 3.2 Expectations from joining a walking group

**Image 3:**
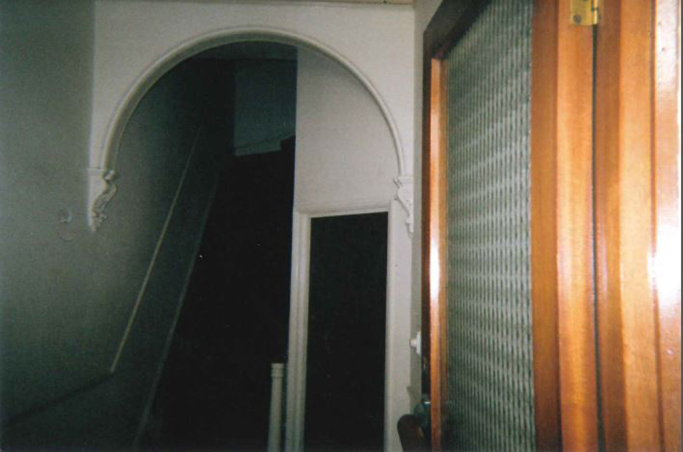
Breathlessness on the stairs was a trigger to seek medical help.

Referred by their family doctor, most participants had multiple health problems. Amongst the physiological symptoms, breathlessness was commonly mentioned as a way of judging poor health and fitness. This was illustrated by a photograph (Image 3) from ‘Robert’ who explained that breathlessness and an inability to climb the stairs was a trigger to seeking medical advice.

The walking group had not been actively sought out by any participant as a solution to their health problems but rather as the preferred option to a gym. For some this was an opportunity to try something new and for others it may have been regarded as the ‘least worst’ option.

*No I couldn’t do the gym that is a stretch too far that one. (Brenda)*

*Just something different isn’t it, got fed up with the gym thought I’d do something different so I picked the walking group. (Sharon, aged 40–42)*

Despite making this choice, the walking group was generally not viewed as offering purposeful exercise prior to joining. Although health goals, such as weight loss, were set in conjunction with the trainer, none came to the scheme with high expectations that the walking group would help. Largely, health goals were explained in general terms, such as, ‘getting health back’, ‘feeling better’ or more specifically, ‘losing some weight’. A sense of achievement from endeavouring to do something also seemed as important as any anticipated improvement.

*I thought, walking, is that really going to help that much but I thought I’d give it a go. (Tracy)*

*I thought I might be able to get something if I can walk fast and get some kind of exercise. (Robert)*

### 3.3 Motivators to maintaining membership of the walking group scheme

Despite reservations prior to joining, participants continued with the scheme and attended regularly. Although there had been little expectation, they reported positive experiences. This was expressed as embodied changes such as less breathlessness, better wellbeing and enjoyment of the activity for its own sake. The walking group became a purposeful activity with multiple benefits. Health benefits were couched in examples of impact on everyday living and appeared to be the motivator for continuing with the group walks. Despite the changes experienced, surprise was expressed that a walking group had impacted their health.

The predominant physiological change was a reduction in breathlessness. Participants used this as a barometer of health improvement as well as comparing it with their previous lack of walking ability.

*At the beginning I was getting a bit puffy when we walked fast but that has improved. (Carol, aged 75–80)*

*By the time we got back I was sort of, really out of breath and I really, really felt it. But now, … I am walking and talking and I can see that I can walk quicker. (Liz, aged 40–45*)

In combination with reduced breathlessness, weight loss was another motivator for continuing with the walking group, albeit it in combination with other improved health behaviours.

*It’s helping me with losing weight… my trousers now are either loose or fit properly instead of being tight. (Liz)*

Manifestations of improved wellbeing and psychological health were also frequently expressed and appeared to have become motivators. For example, feeling more energised and confident and also a sense of accomplishment. The use of the natural environment was seen to facilitate this improvement. It was a viewed both as ‘getting away’ and also a calming experience in itself. It was seen as something to look forward to with the positive effect felt into the time after the walk.

*It’s surprising, I feel a lot more energised, whether, I don’t know what it is, you do exercise and you feel energised. I don’t get that bit. (Liz)*

*Happier, content, more content at peace. All the above, sort of thing. (Carol)*

*It gives me a time to think, if I’m stressed it calms me down and focus and I feel better after I have done it. (Jackie)*

Flora, fauna and vistas were photographed by all and discussed during the interviews. Walking in the natural environment was what made it ‘*a nice walk*’ for some. Others expressed it more reticently, such as *‘I suppose you could call it the nature side of things’* (Brenda). The outdoor spaces (beach and park) used for these group walks has many urban features. However, these were not commented on negatively. The environment used was seen as a good walking experience because of the wide open spaces. This enabled participants to stride out ahead of others and personalise their walks as they saw fit.

*Out walking by the sea or in the country, it takes on a different atmosphere and focus and also you don’t realise while you are taking in the scenery just how far you are walking. (Jackie, aged 60–65)*

Finally, participants felt that the walking group facilitated their walking ability, which they translated into everyday walking. Surprise was expressed about how strenuous the group walk was, in terms of both pace and distance. This was especially noticed at the first week’s attendance. They all felt they walked both further and faster due to the influence of others. They observed the speed of others and judged their improvement by comparison. The presence of a leader to set the route but then being able to individualise the walk was seen as important. Distance was increased by adding switch back routes; more difficult terrain was actively sought out by some to add challenge and pace was increased to form deliberate speed work during a group walk. The use of a leader to oversee the group but also encourage individuality was seen to directly contribute to this.

*I think when you walk in a group, you walk at a pace and you have got other people to keep pace with and instead of dawdling along and meandering around you push yourself because somebody else that can walk quicker. (Liz)*

*Yes, I’ve started walking faster. I used to be at the back and now I’m in the middle and I want to be right up the front. (Carl, aged 65–70)*

*I walk further with the group, walking with people is definitely better because as I say after about 20  min I would think I am going home now but there are other people there and if you say, I am going home now, they would think wimp* . *(Brenda)*

One participant specifically mentioned how he had extended the route and increased the pace and challenge to get maximum benefit.

*I did consider stopping the group because by keeping with the group, purely with the group, to a degree it wasn’t pushing me enough, in my opinion, which is why I loop off, loop back and look for the harder terrain sometimes and things like that. (Peter, aged 65–70)*

There was pleasure expressed at both re-discovering walking and also improving walking ability. This impacted on walking in everyday life, both for leisure and for transport.

*These (walking) boots I bought, 3 or 4 year ago and they’ve sat on a shelf 2 years. I haven’t had a chance to go walking on me own… now have confidence to walk on my own. (Mark, aged 60–64)*

*Before I started walking with the group I’d get the bus into town and the bus back whereas now sometimes I’ll walk in one way and get the bus the other way or I’ll walk both ways. When I first walked it, it took me 45 min, it now takes me 25 min. So that is how much fitter and quicker I’ve got. (Tracy)*

### 3.4 The social aspect of the group

The role of the group itself and the social aspect of walking together, was experienced and articulated in complex ways by the participants. All participants joined the walking group separately and none had met before. Anxiety was expressed about joining and meeting others, and the first session was anticipated as a hurdle to be crossed. There was general relief that no-one wore fitness clothing and that others looked as they did in all ‘shapes and sizes’. Generally, participants had limited financial means and did not purchase walking gear such as wet weather clothing, poles and walking shoes, as might be seen in other walking groups. As well as getting used to the exercise, participants described that they had to also get used to the group.

*When I first started I thought I’m not sure how I am going to get on with these different folk but they all seem to have got together, very much so. You walk with people and you bring something out in them as you walk. (Carol)*

Aside from some initial concerns about social interactions and possible awkwardness, some participants welcomed the opportunity for social interaction. The group enabled companionship or simply some distraction from the strain of walking.

*To meet people… I do like talking to X, yeh, makes you feel better, gets you going. (Sharon)*

*I don’t go out anywhere so I get the social aspect, walking with them, talking with them while I am walking which then unconsciously I am walking faster because I am talking to somebody and I don’t notice I am getter faster and speeding up. (Tracy)*

*It’s helped because I am walking along talking to everybody on Tuesdays, having a laugh and that, so as I say someone to talk to. (Carl)*

**Image 4:**
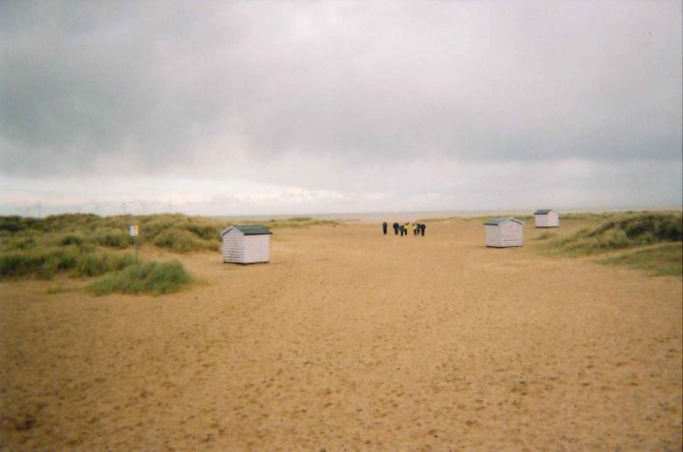
This participant felt “you don’t need to be in the ‘thick of the group’”.

Participants also realised that they could ‘opt out’ of the social aspect of the walking group and that walking with others did not necessarily mean socialising with them. This facility to walk separately, creating space between themselves and the group but with the presence and in sight of others was frequently as expressed as an important feature of the psychological benefits of the group walks, even amongst those who enjoyed the more social aspects. Although the person on their own is not far behind others in the group in this particular Image 4, one participant (Brenda) used it to express that you did not need to be in the ‘thick of the group’.

*I don’t actually get too much involved with the group, I am quite happy to walk on my own, it is also nice to know that there are people there. If I wanted to have a chat I could speed up or hang back. Together even if they are apart, you might have one on the end who is not talking to anybody but they are there. (Brenda)*

Some participants continued to regard the group aspect of the walk as the least attractive part of the format; they simply saw the walking group as a functional way to exercise effectively.

*I am not very good at mixing with people, I never have been. When I go I want to do the walk and to get it done and get some exercise out of it. Not really to associate, just to get fit. (Robert)*

*The group thing is not too important to me personally. I can understand it being important for some people who are lonely and the need to socialise… but I am perhaps not the most sociable of people, I don’t know. That’s not part of the motivation for me, the motivation is keeping fit and exercising and that’s a means to an end. The fact that it is an organised activity gives me the get up and go to get up and do it. I am happy to be within the confines of the group because that gives me the motivation and the regime to work to and to attend. (Peter)*

Overall, it was noteworthy that the social aspect of the group did not predominate during the interviews. However, the presence of others in a group format might have sustained involvement, helped to form habits and stretched personal goals and therefore the group aspect was important.

*Sometimes you start off with good intentions on your own but you don’t really follow through and then you don’t really know if you are pushing yourself enough. (Jackie)*

*It is a regime and you’ve got dedicated times makes me more inclined to go the fixed regime that you have a walk at this time. (Peter)*

This could also be explained by a shared sense of purpose afforded by the group. Whilst the goals were unique to each participant, they had all joined through the same referral route with a shared understanding that the aims of the walking group were to improve health. For example, during the walks there was much sharing of health information; weight loss; medication reduction; reduced breathlessness; perceived healthy foods.

*We get on with what we’ve got to do, and that’s get fit and healthy and that. Yeah, it’s a nice sociable group. (Mark)*

*I don’t think I particularly need the company as much as I need the exercise. I thought it would possibly be the social side but I don’t think I particularly need that. (Brenda)*

## 4 Discussion

Our study explored expectations and experiences of participating in an outdoor walking group as part of a referral scheme. Most were unfamiliar with walking groups and had low expectations of what it would do for their health and wellbeing. Participants captured images of a variety of walkable physical spaces but walking was not expressed as a form of exercise and the walking group was not expected to be purposeful exercise either. Despite prior reservations, people continued with the scheme supported by positive experiences, and reported a developing awareness of their improved health and wellbeing and some enjoyment in the activity for its own sake. Most importantly it had become a purposeful activity with health benefits. The sense of shared purpose and achievement of health goals was a more dominant aspect of the group format than socialising.

The health benefits of group walking were not well understood by our participants before starting the group. This replicates previous findings regarding the misconception about walking not being proper exercise (Darker et al., 2007). There is also an issue of a ‘no pain, no gain’ approach that fails to appreciate walking as exercise (Ekkekakis et al., 2008). This undermines group walking as a useful option to those promoting the benefits of increased physical activity. As found in previous research, our participants also believed their physical activity levels to be satisfactory (Croker et al., 2012). On joining the group they were surprised at how physically demanding a walking group actually was, and accordingly, how unfit they were. Additionally, there was a lack of perception of the links between a lack of exercise and chronic conditions, such as the breathlessness from obesity and poor fitness, which creates a barrier to physical activity behaviour change (Everson-Hock et al., 2013).

In terms of the social environment created by the group format, this study can add to a growing body of literature that investigates the social influences on participation in physical activity. There may be an intuitive appeal that group based interventions are attractive due to their inherent social interactions and indeed some walking groups are successfully marketed as a social way of walking and meeting people (Walking for Health, 2015). For this walking group, the activity became a shared practice, a working group, task oriented around health goals, unlike, for example, a support group where social cohesion is of primary concern (Hoddinott et al., 2010). Despite what is a modest amount of time committed together as a group and lack of prior social networks, the participants identified with others in the group around health improvement goals.

Our finding of group identification around health improvement goals somewhat supports previous research which found that rather than the volume of social contact, it is the number of group identifications that supports healthier behaviour (Sani et al., 2014). It may be because identifying with the group affords a sense of structure and meaning with positive social relationships based on trust and support (Sani et al., 2014, Sani et al., 2012). This has also been found in research with people with depression, finding that group-based interventions were most effective when patients identified with the social group in question and that, ‘it is not groups per se that cure depression, but rather groups with which we identify that cure depression’ (Cruwys et al., 2014, p. 145). However, while our participants shared a common health goal, they did not participate in the group because of wider shared interests, for example, enjoying walking for leisure. They had entered the group as part of a referral scheme, and at best shared a dislike of the alternative referral option, joining a gym. The participants in this study were at best ambivalent about sociability and were not attracted by the social aspect of the group. Rather, it was seen rather as something to be navigated and there was apprehension about joining and becoming part of a group. For those who were of low mood and with enduring mental health problems apprehension of the expectations of sociability in a group format represented a significant barrier. This would support findings from a recent systematic review on barriers to participation in physical activity in older people which highlighted that social awkwardness, such as apprehension of social situations could act as a barrier to group-based activities (Franco et al., 2015).

Our findings show that complex mechanisms seem to be at play when understanding walking groups; social context and influences can act both as barriers and facilitators and these might be intertwined. Having joined the group, our participants valued having time by themselves during the walk time, separate to the group. This was particularly apparent with those of low mood and enduring mental health issues who appreciated the presence of others but valued walking alone, to be free from conversation and the burden of socialising with others. It may be that walking groups such as this, organised with natural pauses, breaks into single file, and low eye contact, benefit the wellbeing of those who find social interaction difficult and they become a temporary social place which may be experienced as restorative (Doughty, 2013). This has been expressed in other walking and therapeutic landscape research as, ‘walking with’, a temporary enactment of companionship with supportive moments of silence without feeling socially awkward (Doughty, 2013). For others, looking for physical challenge the group aspect allowed comparison with others from which to compare their own improvement. They valued the structure of the walk but did not want to be constrained by the pace of others. Therefore, for both physical and psychological needs the group format was important but for enabling individuality within a structured format, rather than for sociability.

The exploration of walking in everyday life showed that walking was not necessarily regarded as ‘normal’, i.e. a common or socially acceptable activity by our participants from low socio-economic backgrounds. Walking should not be considered simply an individual or group activity, but a practice with meaning, acceptability and opportunity shared within a social group or class (Blue et al., 2014, Bourdieu, 1980). As Nettleton and Green (2014) suggested in their investigation of cycling as a social action it is both embodied by social actors and embedded in its specific social context, some practices are considered ‘unthinkable’ within particular social worlds. Similarly, our participants experienced the walking group as a process of learning. At first, prior to joining, being sceptical, then getting used to walking as a form of activity acceptable for people with similar social backgrounds, and finally experiencing its health benefit with their bodies. Our participants expressed as a lack of confidence in joining the group and a concern about ‘what others would looked like’ and would wear and this presented a potential barrier to joining. As discussed by Green (2009) the social organisation and experience of walking has not been adequately understood. Green notes that leisure walking is *embodied* because it is the goal itself, not merely getting from A to B. The meaning lies in actually experiencing the sensation of moving. This was somewhat seen in our study where the participants viewed the experience of walking with the walking group as being of purposeful activity in contrast to their views of walking in everyday life which they did not view as useful exercise. The walking group should therefore perhaps be viewed as a different social practice to walking alone.

## 5 Strengths and limitations

A strength of our study was the use of participant generated photographs in the research process. This aided our understanding of the meaning and ‘insider’ experience of place, an important component of constructing health knowledge (Kearns and Joseph, 1993). By using photograph elicitation the participants were more actively engaged from the beginning of the research process and during the interview used the photographs to talk about their experiences on their own terms. Participant generated photographs can act as a way of engaging participants in research and as a communicative bridge when ideas (such as the language of physical activity) are difficult to articulate helping to connect the culturally different worlds of the researcher and the researched (Guillemin and Drew, 2010, Ward et al., 2015). They are also a useful way of aiding rapport and interaction between the researcher and the researched. There was evidence of much care in the planning and capturing of images which generated more considered responses during the interview. The participant-driven nature of the interviews and the inductive analysis enabled us to uncover unexpected findings such as the ambiguous views on the social aspect of the group.

There are limitations to this study. This was a small sample size, limited to the actual walking members who joined the new scheme. Also, the researcher was a known volunteer with the group which appeared to enable rapport and a relaxed and ‘open’ interview but there is the possibility that the researcher may not have been seen as completely neutral. Finally, the participants represented a very homogenous group (white English). This aided saturation in the analysis but further studies, for example exploring experiences of people from black or minority ethnic groups should be conducted.

## 6 Conclusions and implications for practice

This study worked with a walking group in an exercise referral scheme operating in an area of social and socioeconomic deprivation. Our findings suggest that while our participants had negative expectations of the participation in a walking group (being forced into awkward social interaction with limited tangible health benefits), it was the unexpected positive experiences that encouraged them to stay in the group.

Firstly, while health professionals could certainly provide more detailed information about walking as good or sufficient exercise and how these groups operate, it is the actual provision of such opportunities through exercise referral that seems to make the difference. Our participants experienced better health after joining the group and it seemed to be this visceral feeling of improved fitness, health and wellbeing such as reduced breathlessness that motivated them to continue. Secondly, the group format was sustained as a working group for purposeful physical activity with shared health goals, not due to sociability. Promoting walking groups as a social activity for this group of people may well have been counter-productive as not everyone enjoys socialising, in particular not with a group of strangers, as can be the case with referral schemes. It is noteworthy that our participants expressed that belonging to a group did not necessarily mean enforced interaction and this was important to them. Instead they chose to be social when it suited them and as a walking group developed into a working group there was a shared social acceptability of walking between people of similar social backgrounds.

We believe the findings from this study make a contribution to effective recruitment approaches that reflect the needs and expectations of ‘hard to reach groups’ (Foster et al., 2011). They support previous findings (Matthews et al., 2012) that targeted recruitment methods, in our case through an exercise referral scheme, are the most effective way to engage new walkers from disadvantaged groups into walking interventions. They further support the importance of health, and exercise professionals raising awareness of the benefits and low risks of walking to their patients and clients (Hanson and Jones, 2015, Franco et al., 2015).

## Figures and Tables

**Table 1:** Images photographed by participants.

Images depicted	Percentage
(*n*=210)
Manmade vistas (parks, promenades, cemetery)	30
Cars, car parks and roads	22
Countryside view (fields, dunes, seaside)	20
Walking group (people predominating)	13
Nature – represented by flora, fauna, weather	8
Home environment	5
Other	2
Total	100
